# 

**Published:** 1897-02-20

**Authors:** 


### EARLY EDITION.

**Notices of Births, Deaths, and Marriages** are inserted in this column at a charge of 2s. 6d. for an announcement not exceeding four lines.

### NOTICE TO CORRESPONDENTS.

All MS., Letters, Books for Review, and other matters intended for the Editor should be addressed The Editor, "The Hospital," The Hospital Building, 28 & 29, Southampton Street, Strand, W.C.

The Editor cannot undertake to return rejected MS., even when accompanied by stamped directed envelope.

### Notice to Advertisers and Subscribers.

All Advertisements, Orders for Copies of The Hospital, and Business Communications should be addressed to **THE MANAGER, (Not to The Editor),** The Hospital, The Hospital Building, 28 & 29, Southampton Street, Strand, London, W.C.

The Cost of Subscription, post free, is as follows:—Per week, 2½d.; per quarter, 2s. 8d.; per half-year, 5s. 3d.; per annum, 10s. 6d. Foreign:—Per annum, 15s. 2d.; payable, in all cases, in advance.

**NOTICE.—Vol. XX. of "The Hospital" (April to September, 1896), in handsome cloth binding, is now on sale at the Office of this Journal, price 7s. 6d. Cases for binding the Numbers contained in this Volume. 1s. 6d. Index to Vol. XX., 2½d., post free.**

**The Monthly Part for March will be ready on March 1st, Price 6d., by post 8d.**

The great smoking question at the Harveian Society was decided on February 4th against the smokers. Many curious reasons were brought forward against the proposal, among which was the suggestion that "if ladies were ever admitted members of the society" they might not like it! Is this, then, the next suggestion?

### SCRAPS AND GLEANINGS.

It has been decided again this year to hold a fête in aid of the Swansea General Hospital.

Messrs. Meaby and Co. (Limited) have sent 10,000 boxes of Tritikola biscuits as a gift to the Indian Famine Fund.

At the Birmingham Children's Hospital the number of beds have been reduced from 75 to 62, with the object of increasing the cubic space per bed.

The Lord Mayor has consented to preside at the annual Court of the Royal Hospital for Diseases of the Chest, City Road, E.C., on March 15th.

Twenty-five out of 31 parishes have passed resolutions objecting to the proposed building of an isolation hospital for the Martley Union in Worcestershire.

The site for a new branch of the Bristol Dispensary has been purchased in Malago Road, Bedminster. 11,042 patients were treated at the Bristol Dispensary in 1896.

Wigan's Hospital Sunday and Saturday collection has increased from £4,598 in 1895 to £4,803 in 1896, all the items showing an increase except the collection on Hospital Saturday.

The number of the patients treated at the West Kent General Hospital, Maidstone, during 1896, has been much less than the number in 1895. Thus the in-patients numbered 398 against 480, and the out 4,745 against 4,824.

A receiving order in bankruptcy has been recently made against Frederick Mason, Acre Lane, Brixton, and we are requested to say that this firm has no connection with George Mason and Co., manufacturers of specialties for invalids, Chelsea Works, Walham Green.

Eleven hospitals have benefited to the amount of £22 10s. each by the proceeds of the Mellin's Food Art Competition; while the sum of £10 6s. 9d., which was collected in the boxes for the Referee Children's Dinner Fund, has been handedover.

### BOOKS RECEIVED.

J. AND A. CHURCHILL.

"The Feeding of Infants." By Edmund Cantley, M.D.

CASSELL AND CO.

"The Year Book of Treatment for 1897."

KELLY AND CO.

"Kelly's London Medical Directory, 1897."

BAILLIERE, TINDALL, AND COX.

"Aids to Bacteriology." By T. H. Pearman and C. G. Moor.

"Functional Nervous Disorders in Women." By T. J. McGillicuddy, M.D.

"Diseases of Women and Uterine Therapeutics." By H. Macnaughten-Jones, M.D.

"Death and Sudden Death." By Professor Brouardel, translated by F. Lucas Benham, M.D.

THE F. A. DAVIS CO.

"Autoscopy of the Larynx and Trachea." By Alfred Kirstein, M.D.

THE IDEAL PUBLISHING UNION.

"The Medical Side of the Drink Question." By Sir B. W. Richardson.

SAVOY PRESS.

"Musa Medica." By J. Johnston, M.D.

SAXON AND CO.

"Everybody's Guide to Photography."

"Everybody's English Song-Book."

THE SCIENTIFIC PRESS.

"The Menopause and its Disorders." By A. D. Leith Napier, M.D.

### THE STUDENT OF MEDICINE.

### APPOINTMENTS.

C. Bennett, M.R.C.S.Eng., reappointed Medical Officer of Health to the Fairfield Urban District. T. L. Brown, M.R.C.S.-Eng., L.S.A., appointed Medical Officer and Public Vaccinator for the Hoxton District of the Shoreditch Union. W. N. Clemmey, M.R.C S.Eng., appointed Honorary Surgeon to the Bootle Borough Hospital. H. C. Crouch, M.R.C.S.Eng., L.R.C.P.Lond., appointed House Surgeon and Anæsthetist to the Royal Orthopædic Hospital. W. P. T. Daniel, M.R.C.S., L.R.C.P.Lond., D.P.H., appointed Medical Officer for the Kirkburton District of the Huddersfield Union. G. S. Foulds, L.R.C.P.Edin., L.R.C.S.Edin., L F.P.S.Glasg., appointed Assistant House Surgeon to the North Staffordshire Infirmary, Stoke-on-Trent. E. J. Fox, B.Sc. London, M.R.C.S.Eng., L.R.C.P.-Lond., appointed House Surgeon to the Manchester Royal Infirmary. A. O. Honnywill, L.R.C.S , L.R.C.P., L.M.Edin., appointed Medical Officer and Public Vaccinator for the Sutton and Cheam District of the Epsom Union. T. H. Hughes, appointed Medical Officer of Health to the Connahs Quay Urban District Council. S. G. Vinter, M.R.C.S., L.R.C.P.Lond., appointed Medical Officer to the Union House and Medical Officer and Public Vaccinator to the No. 2 District of the St. Germans Union. A. D. Crawford, M.B., C.M.Glasg., appointed Medical Officer and Public Vaccinator for the Maxstoke and Coleshill Districts of the Meriden Union. Dr. R. Daniel, appointed Medical Officer of Health to the Chatteris Urban District Council.

### ROYAL NATIONAL PENSION FUND FOR NURSES.

*President*—H.R.H. THE PRINCESS OF WALES.

*Chairman*—WALTER H. BURNS, Esq. *Deputy-Chairman*—HENRY C. BURDETT, Esq.

PENSIONS. SICKNESS. ACCIDENT.

### Total Invested Funds, £300,000.

Nurses are invited to join the Fund on account of the substantial and exceptional advantages which it offers them and which *they cannot obtain elsewhere.* The following are the chief points :—

1. **The Fund is Mutual and essentially Co-operative.**

2. **Easy Payment of Premiums.**

   Nurses can pay their premiums monthly or otherwise as best suits their convenience.

3. **The Fund is open to every Nurse.**

   Nurses can assure for Pensions of any amount commencing at any age.

4. **An Investment and Savings Bank.**

   Those entering under the returnable scale can have their premiums returned to them with compound interest, less a small deduction for working expenses. Interest allowed on deposits.

5. **Additions to Pensions.**

   Besides the Pension entered for, substantial additions thereto may be anticipated in the shape of bonuses.

6. **Sickness and Accident Assurance.**

   Policies are issued in connection with Pension policies assuring 10s., 15s., or 20s. a week in cases of incapacity from work through sickness or accident.

Full particulars to be obtained from **THE SECRETARY,**
ROYAL NATIONAL PENSION FUND FOR NURSES,
28, FINSBURY PAVEMENT, LONDON, E.C.

